# Supplementary material for: Function of the SNARE Ykt6 on autophagosomes requires the Dsl1 complex and the Atg1 kinase complex
Source: EMBO Rep. 2020 Oct 7;21(12):e50733. doi: 10.15252/embr.202050733 (PMC7726795; doi:10.15252/embr.202050733)
Supplement: Supplementary file 1 — Expanded View Figures PDF [file EMBR-21-e50733-s001.pdf]

Expanded View Figures

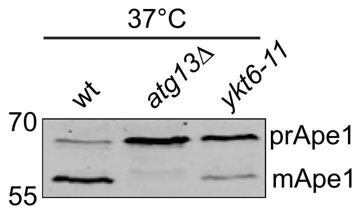

Figure EV1. Ape1 transport in wild-type and mutant cells.

Cells were grown at 24°C to mid-log phase and then shift to 37°C for 2 h. Samples were prepared by TCA precipitation, resolved on SDS gels, blotted to nitrocellulose, and decorated with an antibody to Ape1.

© 2020 The Authors
