# Supplementary material for: Function of the SNARE Ykt6 on autophagosomes requires the Dsl1 complex and the Atg1 kinase complex
Source: EMBO Rep. 2020 Oct 7;21(12):e50733. doi: 10.15252/embr.202050733 (PMC7726795; doi:10.15252/embr.202050733)
Supplement: Supplementary file 2 — Table EV1 [file EMBR-21-e50733-s002.docx]

**Table EV1**. Strains used in this study

| **Strains** | **Genotype** | **Reference** |
| --- | --- | --- |
| BY4741 | MATa *his3∆1 leu2∆0 met15∆0 ura3∆0* | Euroscarf Library |
| SEY6210 | MATalpha *leu2-3 leu2-112 ura3-52 his3-∆200 trp1-∆101 lys2-801 suc2-∆9 GAL* | Reggiori F |
| BJ3505 | MATa *pep4∆::HIS3 prb1-∆1.6R lys2-208 trp1∆101 ura3-52 gal2* | Betty Jones |
|  |  |  |
| CUY325 | BY4741; *ykt6∆::MET VAC8-td-TOMATO::kanMX pRS403-GAL1pr-YKT6-eGFP::GAL1pr* | Meiringer et al., 2008 |
| CUY327 | BY4741; *ykt6∆::MET SNX41-td-TOMATO::kanMX pRS403-GAL1pr-YKT6-eGFP::GAL1pr* | Meiringer et al., 2008 |
| CUY331 | BY4741; *ykt6∆::MET SEC63-td-TOMATO::kanMX pRS403-GAL1pr-YKT6-eGFP::GAL1pr* | Meiringer et al., 2008 |
| CUY851 | BY4741; *ykt6∆::MET MNN9-td-TOMATO::kanMX pRS403-GAL1pr-YKT6-eGFP::GAL1pr* | Meiringer et al., 2008 |
| CUY1193 | MATalpha *ura3 his3 leu2 lys2 trp1 sec20-1* | Dieter Schmidt |
| CUY2367 | BY4741; *ykt6∆::MET pRS403-GAL1pr-YKT6-GFP::HIS3* | Meiringer et al., 2008 |
| CUY9839 | MATalpha *his3∆200 leu2∆0 lys2∆0 met15∆0 trp1∆63 ura3∆0* *ATG1pr::NatNT2 ATG1-TAP::kanMX GAL1pr-ATG13::hphNT1* | This study |
| CUY10048 | BY4741; *vam3∆::kanMX ATG9-3xFLAG::hphNT1* | Gao et al., 2018 |
| CUY10051 | BY4741; *vam3∆::kanMX ATG9-3xFLAG::hphNT1 pRS416-pCuGFP-ATG8::URA*3 | Gao et al., 2018 |
| CUY10171 | BY4741; *vam3∆::kanMX ATG9-3xFLAG::hphNT1 GFP-ATG8::natNT2* | Gao et al., 2018 |
| CUY10447 | BJ3505;*VAC8-3xmCherry::natNT2* | Gao et al., 2018 |
| CUY13332 | SEY6210; *mel-ykt6-12 ATG9-3xFLAG::hphNT1* | Gao et al., 2018 |
|  |  |  |
| CUY11466 | SEY6210; *mel-ykt6-11 pRS416-pCuGFP-ATG8::URA*3 | Gao et al., 2018 |
|  |  |  |
| CUY11469 | BY4741; *ykt6∆::MET pRS403-GAL1pr-YKT6-GFP::HIS3 pRS415-pmCherry-V5-ATG8::LEU*2 | Gao et al., 2018 |
|  |  |  |
| CUY11667 | BY4741; *ykt6∆::MET pRS403-GAL1pr-YKT6-GFP::HIS3* *mCherry-APE1::hphNT1* | This study |
|  |  |  |
| CUY11668 | BY4741; *ykt6∆::MET pRS403-GAL1pr-YKT6-GFP::HIS3* *mCherry-APE1::hphNT1 atg1∆.:natNT2* | This study |
|  |  |  |
| CUY11669 | BY4741; *ykt6∆::MET pRS403-GAL1pr-YKT6-GFP::HIS3* *mCherry-APE1::hphNT1 atg2∆.:natNT2* | This study |
|  |  |  |
| CUY11670 | BY4741; *ykt6∆::MET pRS403-GAL1pr-YKT6-GFP::HIS3* *mCherry-APE1::hphNT1 atg4∆.:natNT2* | This study |
|  |  |  |
| CUY11671 | BY4741; *ykt6∆::MET pRS403-GAL1pr-YKT6-GFP::HIS3* *mCherry-APE1::hphNT1 atg8∆.:natNT2* | This study |
|  |  |  |
| CUY11672 | BY4741; *ykt6∆::MET pRS403-GAL1pr-YKT6-GFP::HIS3* *mCherry-APE1::hphNT1 atg9∆.:natNT2* | This study |
|  |  |  |
| CUY11673 | BY4741; *ykt6∆::MET pRS403-GAL1pr-YKT6-GFP::HIS3* *mCherry-APE1::hphNT1 atg13∆.:natNT2* | This study |
|  |  |  |
| CUY11674 | BY4741; *ykt6∆::MET pRS403-GAL1pr-YKT6-GFP::HIS3* *mCherry-APE1::hphNT1 atg14∆.:natNT2* | This study |
|  |  |  |
| CUY11675 | BY4741; *ykt6∆::MET pRS403-GAL1pr-YKT6-GFP::HIS3* *mCherry-APE1::hphNT1 atg16∆.:natNT2* | This study |
|  |  |  |
| CUY11680 | BY4741; *ykt6∆::MET VAC8-td-TOMATO::kanMX pRS403-GAL1pr-YKT6-eGFP::GAL1pr pRS315-CUP1pr-BFP-APE1:.LEU2* | This study |
| CUY11681 | BY4741; *ykt6∆::MET SNX41-td-TOMATO::kanMX pRS403-GAL1pr-YKT6-eGFP::GAL1pr pRS315-CUP1pr-BFP-APE1:.LEU2* | This study |
| CUY11682 | BY4741; *ykt6∆::MET SEC63-td-TOMATO::kanMX pRS403-GAL1pr-YKT6-eGFP::GAL1pr pRS315-CUP1pr-BFP-APE1:.LEU2* | This study |
| CUY11683 | BY4741; *ykt6∆::MET MNN9-td-TOMATO::kanMX pRS403-GAL1pr-YKT6-eGFP::GAL1pr pRS315-CUP1pr-BFP-APE1:.LEU2* | This study |
| CUY11898 | BY4741; *ykt6∆::MET pRS403-GAL1pr-YKT6-GFP::HIS3* *mCherry-APE1::hphNT1 atg17∆.:natNT2* | This study |
|  |  |  |
| CUY11899 | MATa *his3∆ leu2∆ ura3∆; dsl3-2::HIS mCherry-ATG8::hphNT1* | This study |
|  |  |  |
| CUY11901 | MATalpha *his3 leu2 ura3 trp1; tip20-5 mCherry-ATG8::hphNT1* | This study |
|  |  |  |
| CUY11904 | MATa *his3∆ leu2∆ ura3∆; dsl3-2::HIS pRS416-pCuGFP-ATG8::URA3* | This study |
|  |  |  |
| CUY11906 | MATalpha *his3 leu2 ura3 trp1; tip20-5 pRS416-pCuGFP-ATG8::URA3* | This study |
|  |  |  |
| CUY11908 | BY4741; *ykt6∆::MET pRS403-GAL1pr-YKT6-GFP::HIS3 pRS416-TPIpr-mCherry-ATG8 pRS315-CUP1pr-BFP-APE1::LEU2* | This study |
| CUY12152 | MATa his3∆ leu2∆ ura3∆; *dsl3-2::HIS ATG9-3xFLAG::hphNT1 pRS416-pCuGFP-ATG8::URA3* | This study |
|  |  |  |
| CUY12153 | BY4741; *ykt6∆::MET pRS403-GAL1pr-YKT6-GFP::HIS ATG9-mScarlet::kanMX* | This study |
| CUY12157 | MATa his3∆ leu2∆ ura3∆; *dsl3-2::HIS ATG9-3xmCherry::natNT2 pRS416-pCuGFP-ATG8::URA pRS315-CUP1pr-BFP-APE1:.LEU2* | This study |
|  |  |  |
| CUY12223 | BY4741; *ykt6∆::MET pRS415-TPIpr-mCherry-ATG8 pRS413-GAL1pr-YKT6-eGFP* | This study |
| CUY12224 | BY4741; *ykt6∆::MET pRS415-TPIpr-mCherry-ATG8 pRS413-GAL1pr-YKT6 (S182A)-eGFP* | This study |
| CUY12586 | MATalpha *sec12-1; ATG1-3xmCherry::hphNT1* | This study |
| CUY12590 | SEY6210*; mCherry-Atg8::natNT2 PHO5pr-GFP-Ykt6::URA* | This study |
| CUY12593 | SEY6210*; DSL1-3xmCherry::hphNT1 PHO5pr-GFP-YKT6::URA* | This study |
| CUY12761 | SEY6210*; mCherry-Atg8::natNT2 Dsl3-GFP:: hphNT1* | This study |
| CUY12765 | SEY6210*; mCherry-Atg8::natNT2 PHO5pr-GFP-Ykt6::URA atg1^D211A^:: kanMX* | This study |
| CUY12771 | MATalpha *ura3 his3 leu2 trp1 tip20-5* *Sec63-3xmCherry::hphNT1* *pRS413-GAL1pr-Ykt6-eGFP::HIS* | This study |
| CUY12772 | MATalpha *ura3 his3 leu2 trp1 tip20-5* *Sec13-3xmCherry::hphNT1* *pRS413-GAL1pr-Ykt6-eGFP::HIS* | This study |
| CUY10784 | BY4741; *vam3∆::kanMX ATG9-3xFLAG::hphNT1 GFP-ATG8::natNT2* *pRS413-GAL1pr-Ykt6::HIS* | This study |
| CUY10784 | BY4741; *vam3∆::kanMX ATG9-3xFLAG::hphNT1 GFP-ATG8::natNT2 pRS413-GAL1pr-Ykt6 (S182AS183A)::HIS* | This study |
