# Supplementary material for: Function of the SNARE Ykt6 on autophagosomes requires the Dsl1 complex and the Atg1 kinase complex
Source: EMBO Rep. 2020 Oct 7;21(12):e50733. doi: 10.15252/embr.202050733 (PMC7726795; doi:10.15252/embr.202050733)
Supplement: Supplementary file 3 — Table EV2 [file EMBR-21-e50733-s003.docx]

**Table EV2**. Plasmids used in this study

| **Plasmids** | **Reference** |
| --- | --- |
| pRS416*-pCuGFP-ATG8* | Reggiori lab |
| pRS415*-TPIpr-mCherry-ATG8* | Reggiori lab |
| pRS315-*CUP1pr-BFP-APE1* | Claudine Kraft |
| pRS413*-GAL1pr-YKT6-eGFP* | Meiringer et al., 2008 |
| pRS413*-GAL1pr-YKT6* | Meiringer et al., 2008 |
| pRS413-*GAL1pr-YKT6 (S182AS183A)-eGFP* | This Study |
| pRS413-*GAL1pr-YKT6 (S182AS183A)* | This Study |
| pRS413-*GAL1pr-YKT6 (S182DS183D)* | This Study |
| pRS413-*GAL1pr-YKT6 (S182DS183D)-eGFP* | This Study |
| *pRCC-K- ATG1-near-211aa* | This Study |
